# Pediatric and Adolescent Hepatitis C Care Cascade and Real-World Treatment Outcomes Utilizing an Integrated Health System Specialty Pharmacy Model

**DOI:** 10.1093/jpids/piaf042

**Published:** 2025-05-06

**Authors:** Alicia B Carver, Cori Edmonds, Kristen Whelchel, Ryan Moore, Leena Choi, Lynette A Gillis

**Affiliations:** Specialty Pharmacy Services, Vanderbilt University Medical Center, Nashville, TN, United States; Specialty Pharmacy Services, Vanderbilt University Medical Center, Nashville, TN, United States; Specialty Pharmacy Services, Vanderbilt University Medical Center, Nashville, TN, United States; Department of Biostatistics, Vanderbilt University Medical Center, Nashville, TN, United States; Department of Biostatistics, Vanderbilt University Medical Center, Nashville, TN, United States; Department of Pediatric Gastroenterology, Hepatology and Nutrition, Monroe Carell Jr. Children’s Hospital at Vanderbilt, Nashville, TN, United States; University of Louisville School of Medicine, Louisville, KY, United States

**Keywords:** hepatitis C, pediatrics, direct-acting antiviral, cascade of care, sustained virologic response

## Abstract

**Objectives:**

This study evaluated the Cascade of Care (CoC) and real-world efficacy of direct-acting antivirals (DAAs) for hepatitis C treatment in pediatric and adolescent patients utilizing an integrated health system specialty pharmacy (HSSP) to assist with medication selection, insurance approval, swallowing practice, initiation, and monitoring.

**Methods:**

This single-center, retrospective, observational cohort study included chronic hepatitis C patients <18 years old evaluated by pediatric hepatologists at an academic health system between January 1, 2017 and September 30, 2022. The primary endpoint was sustained virologic response (SVR) ≥12 weeks following treatment completion in patients initiating DAAs. Secondary endpoints were CoC advancement, variables impacting DAA initiation, patient-reported side effects, and adherence. An ordinal logistic regression model assessed whether initiation time was associated with a patient’s ability to swallow, prior authorization outcome, and medication availability to HSSP at referral. The odds ratio reflects the likelihood of a shift in time to initiation for a given group relative to its respective reference group.

**Results:**

Of 98 patients evaluated by a hepatologist, 73 (75%) were referred to the HSSP. Loss to follow-up was the primary reason (88%; 22/25) for nonreferral, most commonly (73%; 16/22) in those aged ≤5 years. Following HSSP referral, 88% (64/73) initiated DAA and 92% (59/64) of those achieved SVR. Time from HSSP referral to medication initiation was impacted by DAA availability (OR = 41.47; 95% CI, 9.51-180.87; *P* <.001) and inability to swallow the dosage form at evaluation (OR = 3.94; 95% CI, 1.56-9.98; *P* =.004). Over half (53%; 34/64) of patients initiating DAA reported ≥1 adverse event; none discontinued treatment. Most (69%; 44/64) reported no missed doses.

**Conclusions:**

The largest CoC drop-off occurred following initial clinic evaluation, primarily in children aged ≤5 years. Enhanced linkage to care efforts is needed in these patients. Conversely, nearly all patients referred to the HSSP were initiated on DAA, completed therapy, and achieved SVR.

## INTRODUCTION

In 2013, the advent of all-oral direct-acting antivirals (DAAs) revolutionized treatment for adults with Hepatitis C Virus (HCV). However, DAAs were not approved in adolescents aged 12-17 until 2017 (ledipasvir/sofosbuvir [LDV/SOF]) and the first pangenotypic DAA was not approved in this age group until 2019 (glecaprevir/pibrentasvir [GLE/PIB]).^1-4^

The year 2019 also heralded the expansion of LDV/SOF approval for use in children aged 3-11 years, bringing to market pediatric tablet and pellet formulations which were unavailable until 2020 because of production delays and the COVID-19 pandemic.^5-7^ Between 2020 and 2021, sofosbuvir/velpatasvir (SOF/VEL) and GLE/PIB received FDA approval for children as young as 3 years, introducing a new pediatric tablet strength for SOF/VEL and pellet formulations for both.^8-11^ These new formulations experienced production delays similar to LDV/SOF.

Despite years of DAA availability and the AASLD/IDSA recommendation to treat children of all genotypes ≥3 years of age, there is a paucity of data describing real-world experiences in the United States.^12^ Previously, in US HCV-infected adults, barriers to medication accessibility have been identified, including affordability and insurance criteria limiting DAA approvals to those with advanced liver fibrosis, drug and alcohol abstinence, drug and alcohol rehabilitation, and DAAs prescribed by specialists.^13,14^ Current real-world studies in those aged <18 years are limited to regions outside of the United States, which do not consider medication accessibility.^15-18^ Examining the Cascade of Care (CoC) in patients under 18 years could identify unique barriers to DAA accessibility and treatment completion.

Since no liquid DAA formulations exist, patients unable to swallow tablets or pellets may require coaching for medication administration. Crushing or chewing tablet and pellet formulations produces a bitter taste which may induce gagging or vomiting and make subsequent dosing challenging.^7,16,19^ Studies have attributed administration difficulty to early DAA discontinuation, lower adherence rates, and SVR rates as low as 83%.^6,15,20,21^ Recently, the European Society for Pediatric Gastroenterology, Hepatology and Nutrition (ESPGHAN) recommended a swallowability assessment before DAA initiation and subsequently postponing DAAs for those unable to swallow the available formulation.^20^

This study aims to describe the CoC, identify barriers to completion, and assess the real-world effectiveness of DAAs in pediatric patients treated at a US tertiary academic medical center utilizing an integrated Health-System Specialty Pharmacy (HSSP) model.

## METHODS

### HSSP Model

**Figure 1.**
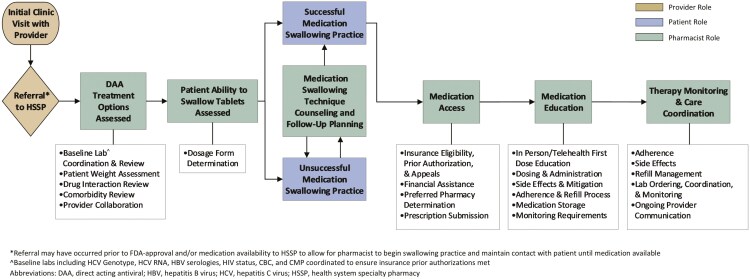
Integrated HSSP Model for Treatment of HCV. Patients are evaluated in clinic by the hepatologist following referral to the Pediatric Gastroenterology, Hepatology and Nutrition Clinic at Monroe Carell Jr. Children’s Hospital at Vanderbilt for HCV care. A referral to the HSSP via the EMR is placed for DAA treatment at the discretion of the hepatologist. Once home administration practice is successful or if the patient can swallow tablets at the time of initial clinic visit, the pharmacist submits for DAA approval through the patient’s insurance provider. All patients receive education and follow-up by the pharmacist.

The Pediatric Gastroenterology, Hepatology and Nutrition Clinic at Monroe Carell Jr. Children’s Hospital at Vanderbilt is a hospital-based, outpatient clinic providing comprehensive hepatology services upon referral. Pediatric hepatologists collaborated with the HSSP in 2017 to establish an integrated model to treat hepatitis C patients (Figure 1). The HSSP team includes 2 full-time pharmacists and 1 full-time pharmacy technician who also manage adult hepatology patients taking specialty medications, including those with HCV. This pediatric collaboration accounts for roughly 5%-10% of overall HSSP hepatology volume.

Referral to the HSSP following clinic evaluation, labs, and fibrosis assessment is at the hepatologist’s discretion and does not require fibrosis assessment or the patient to be DAA-eligible (defined as aged ≥3 years or FDA approval). If ineligible for DAA, the pharmacist completes swallowing coaching and follow-ups as described below until eligible. Fibrosis is assessed most commonly by calculation of APRI score in conjunction with abdominal ultrasound with shear-wave elastography, which is coordinated with the clinic visit at the on-site hospital.

The pharmacist workflow following HSSP referral can be seen in Figure 1. Pharmacists contact patients unable to swallow tablets at the initial clinic visit to discuss methods for swallowing practice and general administration techniques (Table S1). The pharmacist and caregiver follow-up by phone until the caregiver verbally confirms the patient’s ability to swallow successfully without crushing or chewing candy 3 consecutive times. Follow-up frequency is determined by the pharmacist and caregiver. Direct-acting antivirals are dosed by weight in accordance with their package inserts. Ledipasvir/sofosbuvir and SOF/VEL pellets are dosed as one packet daily in a nonacidic soft food and administered within 30 minutes of preparation without chewing.^7,16^ Glecaprevir/pibrentasvir pellets are dosed as 3-6 packets daily in low-water content food and administered within 15 minutes of preparation without chewing.^19^

Following successful swallowing practice, the HSSP submits for DAA approval through the patient’s insurance. Despite FDA approval for a 12-week regimen of LDV/SOF in patients aged <18 years, both commercial insurances and Medicaid often only approved 8 weeks of treatment early in the study period (ie, partial insurance approval), mirroring FDA approval for subsets of adults.^7^ To ensure pediatric patients received the recommended 12 weeks of treatment, insurance appeals were frequently necessary. Tennessee Medicaid updated its clinical criteria in 2021, eliminating the need for such appeals. Additionally, Tennessee Medicaid imposed approval restrictions often not required by commercial plans, including a fibrosis requirement of ≥F2 (defined in Table S2) and ≥6 months of alcohol and substance abstinence, with documentation of rehabilitation completion. These restrictions were in place until January 1, 2019 and March 1, 2022, respectively.^22,23^ If insurance denial occurs, the pharmacist appeals until DAA approval, obtains financial assistance if needed, and submits the prescription and subsequent lab orders for provider review.

All patients and caregivers are educated by the pharmacist in clinic or via telemedicine (Figure 1). Patients receive medication prior to initial education, allowing for first-dose observation. Afterward, caregivers are responsible for ensuring medication administration and adherence.

Following DAA initiation, the pharmacist contacts the patient caregiver by phone at least monthly. Laboratory monitoring, including HCV RNA, occurs at week 4 and again ≥12 weeks after DAA completion, aligning with the patient’s posttreatment in-person clinic visit or telemedicine appointment. During the study period, a Collaborative Pharmacy Practice Agreement was implemented, allowing pharmacists to order prescriptions and labs under hepatologist supervision, eliminating the need for provider review. All patient contact is documented in the electronic medical record (EMR), and providers are notified of any concerns and lab results.

### Design and Endpoints

This single-center, retrospective, observational cohort study included patients ≤18 years old with a diagnosis of chronic hepatitis C (ICD10: B18.2) evaluated by a pediatric hepatologist at the Pediatric Gastroenterology, Hepatology and Nutrition Clinic at Monroe Carell Jr. Children’s Hospital at Vanderbilt between January 1, 2017 and September 30, 2022. Patients were excluded for lack of confirmatory hepatitis C testing, enrollment in a clinical trial, or aged <3 years at the time of study conclusion. Primary endpoint was sustained virologic response (SVR), defined as an undetectable HCV RNA ≥ 12 weeks following treatment completion, in patients initiated on DAA treatment. Secondary endpoints included achievement rates of each stage in the CoC, reasons for nonprogression through the CoC, time from HSSP referral to DAA initiation, variables impacting DAA initiation, patient-reported side effects, and adherence. Time from HSSP referral to DAA initiation was further ordered as time from HSSP referral to successful swallowing practice to medication insurance approval to DAA initiation.

### Data Collection

This study was approved by the Vanderbilt University Medical Center Institutional Review Board (#222292). Data were retrospectively collected and stored in Research Electronic Data Capture (REDCap) hosted at VUMC.^24,25^ Baseline demographics including age, race, gender, weight, and insurance type were collected for all patients. Additional baseline data including HCV genotype, HCV RNA, aspartate aminotransferase (AST), alanine aminotransferase (ALT), cirrhosis status, previous HCV treatment history, concurrent medications, and prescribed DAA regimen were collected for patients initiating DAA. Study definitions are found in Table S2.

### Data Analysis

Patient characteristics and treatment outcomes were summarized using quartiles for continuous variables and counts and proportions for categorical variables. Univariate analysis for continuous variables was performed using Wilcoxon rank-sum tests. In patients initiating treatment, an ordinal logistic regression analysis was performed to examine whether time from HSSP referral to DAA initiation was associated with the ability to swallow at initial clinic evaluation (yes vs no), prior authorization outcome (approved vs denied vs partial approval), and medication available to HSSP at time of referral (yes vs no). The proportional odds assumption for an ordinal regression model (also known as proportion odds model) was also tested.^26^ An ordinal regression model was used to address the highly skewed nature of the outcome.^27^ This approach extends the Wilcoxon rank-sum test, a commonly used nonparametric method, by enabling the inclusion of covariates, making it less sensitive to violations of distributional assumptions. Ordinal regression is particularly useful when the primary interest is in detecting shifts in the distribution of the outcome across levels of a covariate. For example, it can assess whether the time to medication initiation is shorter (ie, shifted toward shorter) for patients who can swallow compared with those who cannot. In this model, an odds ratio greater than 1 indicates increased odds of a longer time to medication initiation for a given group relative to its reference group (eg, patients unable to swallow have increased odds of a longer initiation time compared with those able to swallow). Analyses were conducted using the R programming language, version 4.4.1.^28^

## RESULTS

### Cascade of Care

Ninety-eight patients were evaluated in the clinic and included (Figure S1). The median age was 6 years, with the majority being White (71%) males (51%) insured by Medicaid (84%) (Table S3).

**Figure 2.**
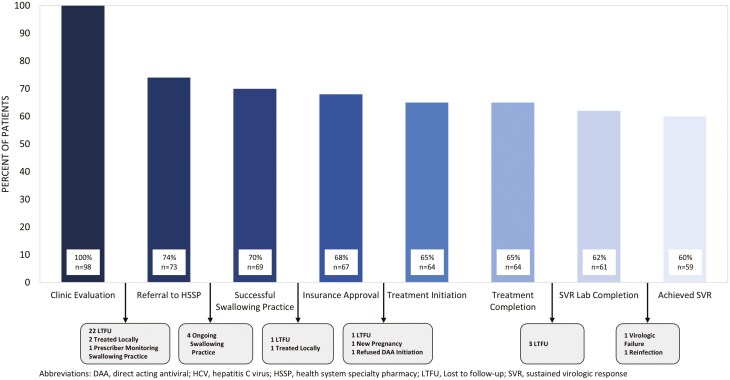
Bar Graph Depicting the Hepatitis C Cascade of Care and Reasons for Gaps Following Each Stage. Of the 98 patients completing clinic evaluation, 73 (76%) were referred to the integrated HSSP, 69 (72%) successfully swallowed the practice dosage form, 67 (70%) received insurance approval, 64 (67%) initiated treatment, 64 (67%) completed treatment, 61 (64%) completed SVR labs, and 59 (62%) achieved SVR.

Most (76%; *n* = 73) of the 98 patients evaluated in the clinic were referred to the HSSP (Figure 2). Of the 25 patients not referred, 88% (*n* = 22) were lost to follow-up (LTFU) after clinic evaluation. Half (50%; *n* = 11) of those LTFU were aged <3 years, 5 (23%) were aged 3-5, 5 (23%) were aged 6-11, and 1 (5%) was aged 12-17.

Following HSSP referral (*n* = 73), 4 (5%) patients did not advance through the CoC because they were unable to successfully swallow the practice dosing before study conclusion but remained engaged with the HSSP. Following successful swallowing practice (*n* = 69), 1 patient was LTFU and 1 patient was treated by the outside provider. Following DAA insurance approval (*n* = 67), 1 patient was LTFU, 1 became pregnant, and 1 refused to attempt DAA administration despite successful home practice; none of these patients initiated DAA therapy. Of the 73 patients referred to the HSSP, 88% (*n* = 64) were initiated on treatment and all completed the prescribed treatment course. Following DAA completion (*n* = 64), 3 (5%) were LTFU, not completing SVR laboratory testing.

### Factors Impacting Time From HSSP Referral to Treatment Initiation

**Figure 3.**
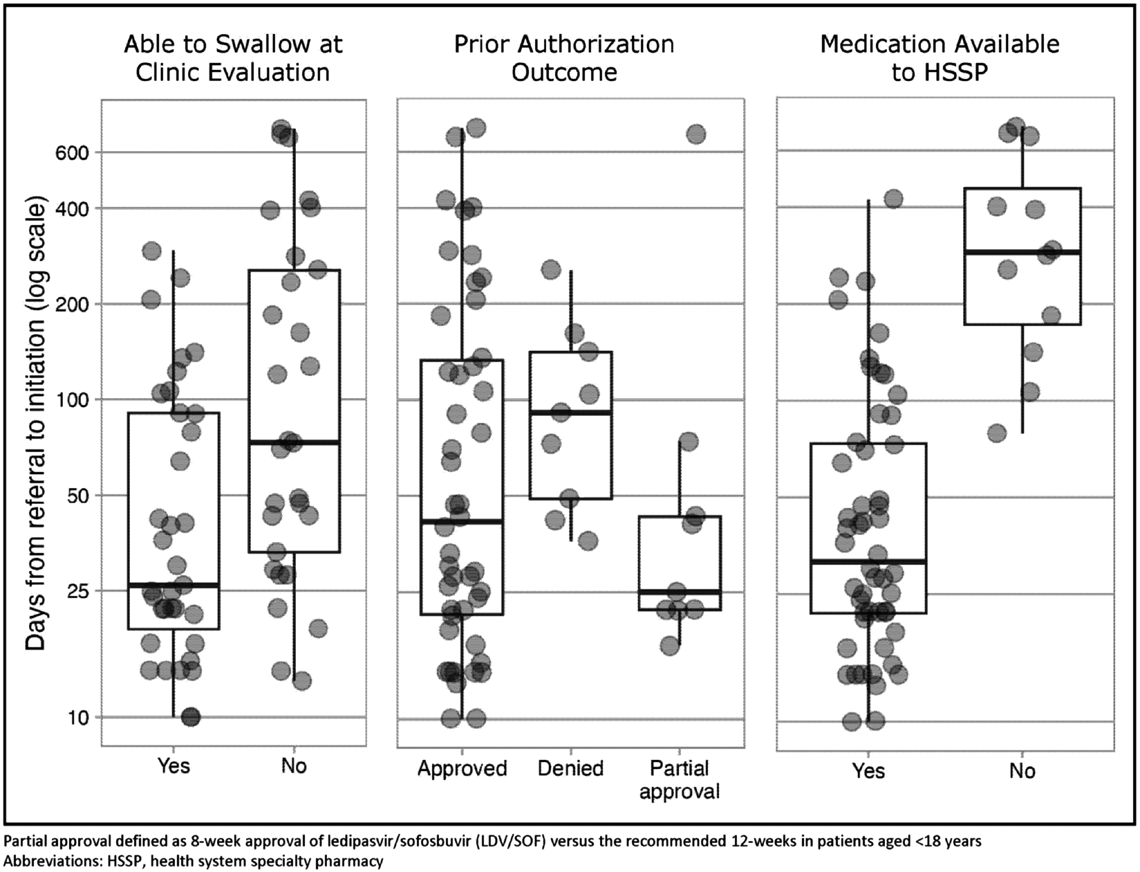
Univariate Analysis Illustrating That Both Patients Unable to Swallow the Practice Medication at Clinic Evaluation (*P* = .004) and Those Patients Whose DAA Was Not Available to the Pharmacy (*P* < .001) Were Significantly More Likely Have Delay in DAA Initiation, While Those Patients That Were Initially Denied DAA (*P* = .112) or Only Partially Approved (*P* = .918) Were Not More Likely to Have a Delay in DAA Initiation.

Times between the CoC stages for patients who initiated treatment (*n* = 64) were broken down by age group and depicted in Table S4. Overall median time from HSSP referral to treatment initiation was 43 days (IQR 22-129). In the univariate analysis, patients who were able to swallow at the initial clinic visit had a significantly shorter time to DAA initiation compared with those who were unable to swallow (26 days [IQR: 19–91] vs 73 days [IQR: 33–255]; *P* =.003). Similarly, if the DAA was available to the HSSP at the time of the patient’s referral, patients experienced a significantly shorter time to initiation compared with those without access (32 days [IQR: 22–74] vs 289 days [IQR: 173–468]; *P* <.001) (Figure 3). In the ordinal regression analysis (Figure S2), when the DAA was not available to the HSSP, patients had 41 times greater odds of having a longer time to first dose compared with those who had medication available (OR 41.47; 95% CI, 9.51-180.87; *P* <.001). Patients unable to swallow at the time of the initial clinic visit had nearly 4 times greater odds of having a longer time to treatment initiation relative to patients who could swallow (OR 3.94; 95% CI, 1.56-9.98, *P* =.004). Insurance initial denial of DAA treatment (OR 2.68; 95% CI, 0.80-9.03, *P* =.112) and partial insurance approval (OR 1.06; 95% CI, 0.33-3.42, *P* =.918) did not have a significant association with time to DAA initiation.

### Real-World Outcomes

Ability to swallow at initial evaluation was assessed for all patients referred to the HSSP (*n* = 73) (Figure S3). Baseline characteristics and treatment outcomes of the 64 patients that initiated treatment are shown in Table 1. The median age was 6 years, the majority were White (73%; 47/64) females (56%; 36/64) with genotype 1 (77%; 49/64) HCV, no cirrhosis (98%; 63/64), and all were naïve to previous treatment. Most patients (67%; 43/64) were prescribed LDV/SOF for 12 weeks. Most DAA formulations prescribed were tablets (86%; 55/64).

**Table 1.** Baseline* Characteristics and SVR Rates of Patients Initiating DAA Therapy

	3-5 years *n* = 20	6-11 years *n* = 32	12-17 years *n* = 12	Overall *n* = 64
Median age,^^^ years (IQR)	5 (4-5)	7 (6-8.25)	15 (13.75-15.25)	6 (5-9.25)
Male, *n* (%)	12 (60%)	13 (41%)	3 (25%)	28 (44%)
White, *n* (%)	14 (70%)	25 (78%)	8 (67%)	47 (73%)
Weight, kg—median (IQR)	18.8 (17.1-20.8)	27.3 (24.8-33.3)	55.7 (43.5-62.4)	26.6 (19.9-38.9)
Genotype, *n* (%)				
1	17 (85%)	22 (69%)	10 (83%)	49 (77%)
2	1 (5%)	2 (6%)	0 (0%)	3 (5%)
3	2 (10%)	8 (25%)	1 (8%)	11 (17%)
4	0 (0%)	0 (0%)	1 (8%)	1 (2%)
Cirrhosis, *n* (%)	0 (0%)	1 (3%)	0 (0%)	1 (2%)
Treatment experienced, *n* (%)	0 (0%)	0 (0%)	0 (0%)	0 (0%)
Baseline HCV RNA, IU/mL - median (IQR)	852,079 (295,219 - 3,668,653)	1,396,356 (423,922 - 2,928,785)	554,147 (349,904 - 2,215,088)	1,077,115 (392,422 - 3,240,835)
Baseline AST, U/L- median (IQR)	56.5 (44.8-72.0)	42 (33.8-54.2)	37 (30.2-50.5)	45.5 (34.8-62.0)
Baseline ALT, U/L- median (IQR)	64 (41.0-88.2)	45.5 (33.8-65.0)	48 (29.8-70.0)	49.5 (34.0-79.0)
Treatment regimen, *n* (%)				
LDV/SOF 90/400 mg × 12 w	0 (0%)	3 (9%)	8 (67%)	11 (17%)
LDV/SOF 45/200 mg × 12 w	13(65%)	16 (50%)	0 (0%)	29 (45%)
LDV/SOF 33.75/150 mg ×12 w	3 (15%)	0 (0%)	0 (0%)	3 (5%)
SOF/VEL 400/100 mg × 12 w	0 (0%)	6 (19%)	0 (0%)	6 (9%)
SOF/VEL 200/50 mg × 12 w	4 (20%)	7 (22%)	0 (0%)	11 (17%)
GLE/PIB 300/120 mg × 8 w	0 (0%)	0 (0%)	4 (33%)	4 (6%)
DAA Formulation, *n* (%)				
Tablets	14 (70%)	29 (91%)	12 (100%)	55 (86%)
Pellets	6 (30%)	3 (9%)	0 (0%)	9 (14%)
Insurance type, *n* (%)				
Medicaid	17 (85%)	26 (81%)	10 (83%)	53 (83%)
Commercial	3 (15%)	6 (19%)	2 (17%)	11 (17%)
Time from Clinic Visit to DAA initiation, median days [IQR]	108.5 [34.2-188.5]	56 [27-260.5]	41.5 [31.8-95.2]	69 [30.5-170.2]
SVR, *n* (%)				
Yes	18 (90%)	31 (97%)	10 (83%)	59 (92%)
No	1 (5%)	0 (0%)	0 (0%)	1 (2%)
Reinfection	0 (0%)	0 (0%)	1 (8%)	1 (2%)
LTFU	1 (5%)	1 (3%)	1 (8%)	3 (5%)

^*^Baseline labs were the most recent labs prior to DAA initiation.

^^^Age at time of DAA initiation.

Abbreviations: ALT, alanine aminotransferase; AST, aspartate aminotransferase; DAA, direct-acting antiviral; GLE/PIB, glecaprevir/pibrentasvir; HCV, hepatitis C virus; IQR, interquartile range; IU, international units; kg, kilogram; LDV/SOF, ledipasvir/sofosbuvir; L, liter; LTFU, lost to follow-up; mg, milligram; mL, milliliters; SOF/VEL, sofosbuvir/velpatasvir; SVR, sustained virologic response; U, units; w, weeks.

Fifty-nine patients (92%) achieved SVR; 3 (5%) were LTFU, 1 (2%) presented at later date with new genotype, and 1 (2%) was a true failure. The true failure occurred in a 4-year-old with genotype 1a, noncirrhotic, treatment-naïve HCV, treated with LDV/SOF pellets for 12 weeks. Although most patients (69%; 44/64) reported no missed doses, the patient who did not achieve SVR reported 13 missed doses. For the 20 patients reporting missed doses, the median number of doses missed was 2 (IQR 1-6). The most common reasons for missed doses were either unknown (30%; 6/20) or due to vomiting (30%; 6/20), followed by difficulty administering the medication (20%; 4/20), forgetfulness (10%; 2/20), alternate caregiver (5%; 1/20), and insurance lapse (5%; 1/20). Patient-reported side effect rates were similar among DAAs (Table S5). Most patients (53%; 34/64) experienced ≥1 side effect, with headache (20%; 13/64) and fatigue (20%; 13/64) being the most common. No patients experienced serious or grade 3-4 adverse events. Drug interactions were minimal (17%; 11/64) and all were managed by the pharmacist, most frequently by holding the interacting medication (55%; 6/11), followed by adjusting the non-DAA administration timing (36%; 4/11) or dose and administration timing of non-DAA (9%; 1/11). Interacting classes of medications were primarily gastric acid-reducing agents (82%; 9/11) followed by oxcarbazepine (9%; 1/11) and herbal supplements (9%; 1/11).

## DISCUSSION

This study reports success utilizing a pharmacist-led program to navigate children and adolescents with HCV through the CoC from referral to the pharmacist for DAA treatment to the achievement of SVR. High DAA initiation rates in patients referred to the HSSP (88%) and high SVR rates in those initiating DAA treatment (92%) were observed. These findings align with similar pharmacy-integrated models in the adult population.^29,30^ Current pediatric HCV studies have focused primarily on earlier stages of the CoC, from perinatal exposure to screening.^31-35^ This study emphasizes the postscreening portion of the cascade, addressing a crucial gap. The highest patient drop-off rate occurred following initial evaluation and was primarily due to LTFU (88%). Among those LTFU, 73% were aged 0-5 years, highlighting the need for enhanced linkage to care efforts for this age group.

Although insurance restrictions and cost have previously been identified as barriers to successful CoC completion and achievement of SVR in the adult population, this study did not find a significant relationship between insurance approval and time to treatment initiation.^13,36,37^ This may be because of the established presence of DAAs on insurance formularies given prior FDA approval in the adult population as well as the HSSP’s proactive management of insurance processes. When patients were partially approved, DAA therapy could be initiated if the appeal process began simultaneously to ensure that appeal was approved prior to completion of 8 weeks of treatment to avoid a lapse in therapy. This finding highlights the value of a resource dedicated to the ongoing management of the insurance approval process.

This study found that 51% of patients referred to the HSSP had difficulty swallowing medication at the initial clinic evaluation. This occurred mostly in younger cohorts aged 3-5 (69%) and 6-11 (55%). Combined, these cohorts required the most pharmacist contacts (97%; 132/136). Additionally, longer times from clinic evaluation to DAA initiation were seen in the youngest cohort (aged 3-5 years), likely due to swallowing difficulty. Patients’ inability to swallow at the initial clinic evaluation significantly delayed time from HSSP referral to DAA treatment initiation. Despite this, all patients who initiated treatment completed the prescribed course, suggesting postponement of DAA initiation until the patient can reliably administer a DAA dosage form. This is supported by a 2024 ESPGHAN recommendation for a swallowability assessment and delay of DAA initiation for those unable to swallow the available drug formulation.^20^ These findings, coupled with other studies attributing early DAA discontinuation, lower SVR rates, and nonadherence to the palatability of the medication, underscore the importance of the initial assessment, management, and ongoing medication administration support during and prior to DAA initiation, particularly in those ≤12 years.^6,15,18^

The success of the integrated HSSP model is likely due to having dedicated, specialty trained staff to navigate medication selection, access, administration, and monitoring. These resources may not be available in all settings. It is also notable that the beginning of the study period was in conjunction with the first DAA FDA approval in patients <18 years. Since then, insurance criteria (eg, fibrosis stage and patient requirements) have become less rigid, making the approval process less complex. Similar models to manage HCV treatment in pediatric populations could be designed utilizing other multidisciplinary approaches, such as occupational therapists, speech therapists, patient support specialists, and nurses to assist patient navigation through the CoC.

High SVR rates (92%) were consistent across all age groups, with only one virologic failure and one reinfection reported. These high efficacy rates align with clinical trials and real-world studies in this population outside the United States.^17,18,38-43^ In clinical trials, patients experiencing ≥1 adverse event were high (70%-87%), with headache being most common (14%-29%) in patients aged 6-17 years and vomiting being most common (14%-27%) in patients aged 3-5 years.^1,3,5,6,8,11^ This higher incidence of vomiting may be due to palatability issues reported in this age group.^6,15,18^ In contrast to clinical trials, this study found that just over half (52%) of patients reported ≥1 adverse event, which was consistent with a recent meta-analysis including real-world studies that found 50%-72% of children and adolescents experienced ≥1 reported side effect.^18^ The most common (>10%) side effects reported were headache, fatigue, nausea, and vomiting, which aligned with other real-world studies.^17,18,38-40,42,43^ This study noted a small incidence (>5%) of sleep disturbances and behavioral changes, which have been reported infrequently in trials.^8,40^ No serious side effects occurred and no side effects warranted treatment discontinuation.

This study had several limitations. Using ICD-10 codes to identify HCV patients may have resulted in underreporting. The study was limited to a single-site academic tertiary medical clinic, focusing only on patients already accessing specialty care via a referral likely following HCV testing. This specialty care is not always readily accessible throughout the United States and, if available, may require long-distance travel. Telemedicine along with the arrangement of local laboratory monitoring were utilized in this study and could serve as a resource for those without access to specialty care. Additionally, the study period included some referrals before DAA FDA approval and before age eligibility. Although this allowed the pharmacist to keep the patient engaged and complete swallowing practice prior to FDA approval, time from referral to approval and initiation was delayed. The COVID-19 pandemic also contributed to some patient LTFU and delays in DAA production post-FDA approval. These latter two limitations were due to the timing of the study and would not be limitations for future studies.

Efforts should focus on improving linkage to care, especially in patients aged 0-5 years. Utilizing an integrated HSSP model for DAA selection, insurance approval, initiation, and management minimized patient loss after referral and achieved high DAA initiation and efficacy rates. Ensuring the ability to administer DAA dosage forms before initiation is crucial for treatment adherence and completion; therefore, DAA initiation should be delayed until this competency is confirmed.

## Supplementary Material

piaf042_suppl_Supplementary_Figure_S1

piaf042_suppl_Supplementary_Figure_S2

piaf042_suppl_Supplementary_Figure_S3

piaf042_suppl_Supplementary_Table_S1

piaf042_suppl_Supplementary_Table_S2

piaf042_suppl_Supplementary_Table_S3

piaf042_suppl_Supplementary_Table_S4

piaf042_suppl_Supplementary_Table_S5

piaf042_suppl_Supplementary_Figure_Descriptions

## Data Availability

The data supporting these study findings are available upon reasonable request to the corresponding author.
