# Supplementary figures and images for: Pediatric and Adolescent Hepatitis C Care Cascade and Real-World Treatment Outcomes Utilizing an Integrated Health System Specialty Pharmacy Model
Source: J Pediatric Infect Dis Soc. 2025 May 6;14(5):piaf042. doi: 10.1093/jpids/piaf042 (PMC12123190; doi:10.1093/jpids/piaf042)

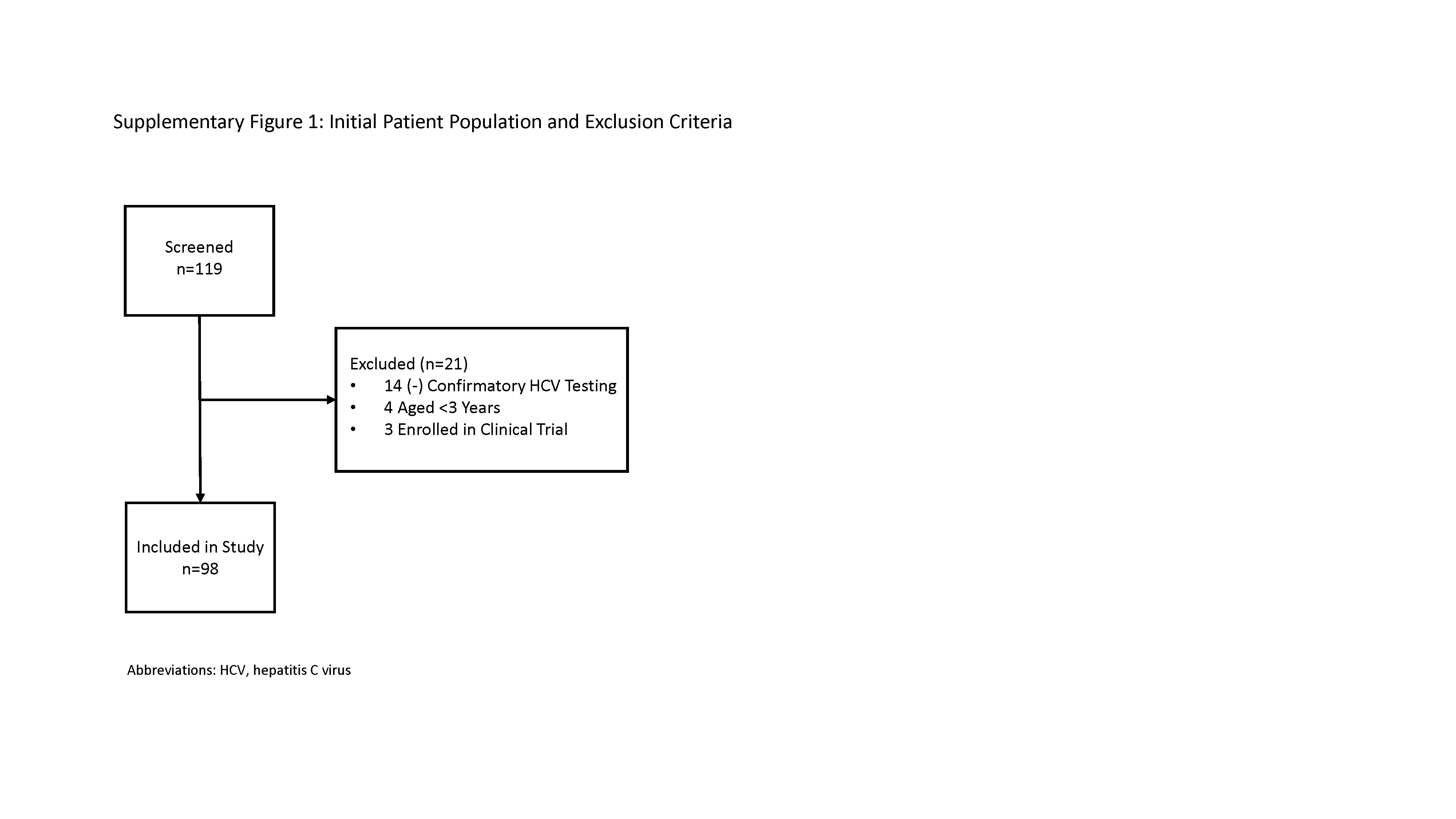

Supplement: piaf042_suppl_Supplementary_Figure_S1 [file piaf042_suppl_supplementary_figure_s1.jpeg]

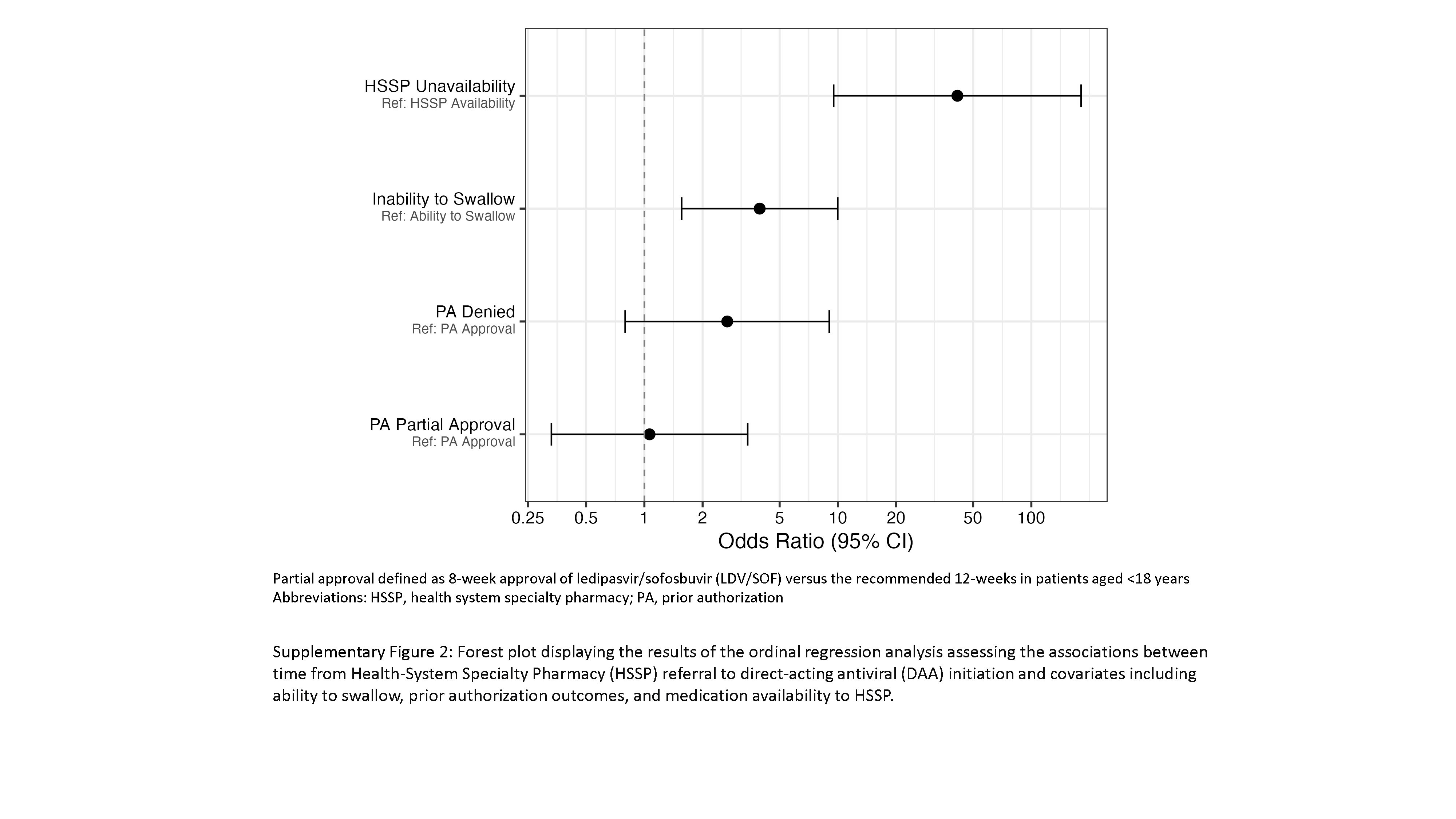

Supplement: piaf042_suppl_Supplementary_Figure_S2 [file piaf042_suppl_supplementary_figure_s2.jpeg]

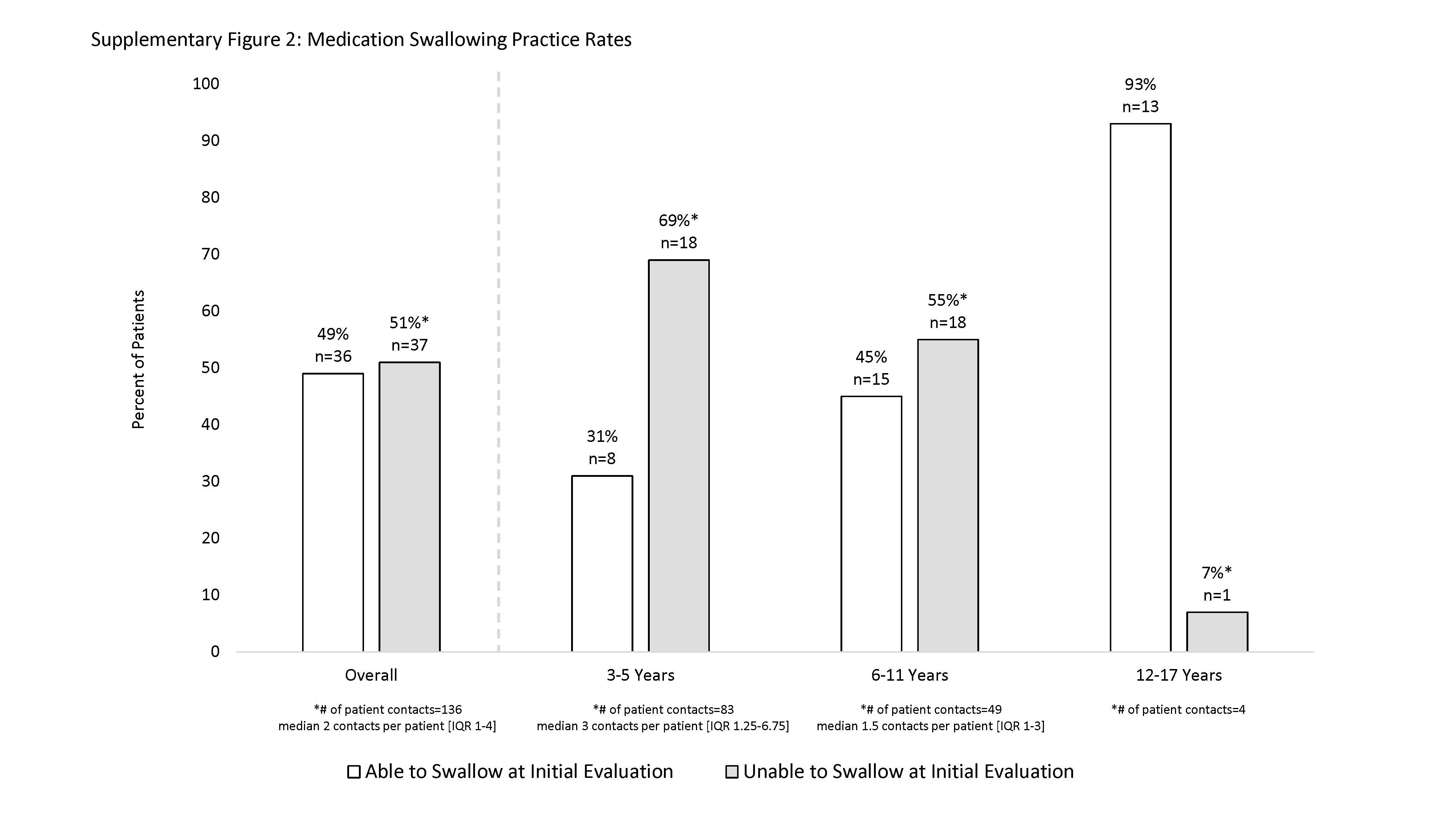

Supplement: piaf042_suppl_Supplementary_Figure_S3 [file piaf042_suppl_supplementary_figure_s3.jpeg]
