# Supplementary material for: Pediatric and Adolescent Hepatitis C Care Cascade and Real-World Treatment Outcomes Utilizing an Integrated Health System Specialty Pharmacy Model
Source: J Pediatric Infect Dis Soc. 2025 May 6;14(5):piaf042. doi: 10.1093/jpids/piaf042 (PMC12123190; doi:10.1093/jpids/piaf042)
Supplement: piaf042_suppl_Supplementary_Table_S1 [file piaf042_suppl_supplementary_table_s1.docx]

Supplementary Table 1: Swallowing Coaching and General Administration Techniques

| **Tablets*** | **Pellets** |
| --- | --- |
| - Practice 5 minutes at a time - Sit straight up in chair - Patient starts with a rod-shaped sprinkle and place on center back of tongue - Take a small sip of water to wash candy down - Increase candy in size incrementally after 3 consecutive successes as follows:   one Nerd 🡪  one Tic Tac 🡪  one M&M 🡪  one Pez   - Pill swallowing cup assist device as an alternative option if practice is not successful - Do not crush or chew candy - Utilize small rewards as incentives - Take long breaks (~days to weeks) before trying again if failure and frustration occur - If tablet swallowing practice continues to be unsuccessful, consider attempting practicing pellets instead | - Practice 5 minutes at a time - Caregiver starts by sprinkling 1-2 Nerd candies on a spoonful of pudding - Have patient swallow without chewing or biting down on candy - Take a small sip of water - Increase number of candies on spoonful incrementally after 3 consecutive successes until approximately 5-10 candies can be swallowed on one spoonful without chewing or biting down - Do not crush or chew candy - Utilize small rewards as incentives - Take long breaks (~days to weeks) before trying again if failure and frustration occur - If pellet swallowing practice continues to be unsuccessful, consider attempting practicing tablets instead if patient’s weight allows |
| *Tablet practice is initially offered if patients were eligible based on weight due to ease of administration. If not  eligible for tablets or if caregiver prefers pellets, pellet practice is pursued. | |
