# Supplementary material for: Pediatric and Adolescent Hepatitis C Care Cascade and Real-World Treatment Outcomes Utilizing an Integrated Health System Specialty Pharmacy Model
Source: J Pediatric Infect Dis Soc. 2025 May 6;14(5):piaf042. doi: 10.1093/jpids/piaf042 (PMC12123190; doi:10.1093/jpids/piaf042)
Supplement: piaf042_suppl_Supplementary_Table_S2 [file piaf042_suppl_supplementary_table_s2.docx]

Supplementary Table 2: Study Definitions

| **Tennessee Medicaid ≥ F2** | Meeting any of the following:   - Metavir score of F2-F4 - FibroTest ^TM^ (FibroSure ^TM^) score of ≥ 0.49 - Ultrasound based transient elastography score of ≥ 7.1kPa - FIB-4 score of >1.45 - APRI score of >0.5 |
| --- | --- |
| **Cirrhosis** | Meeting any of the following:   - Abdominal ultrasound demonstrating anatomical changes consistent with cirrhosis - Liver biopsy with Metavir score F4, FIB-4 score ≥3.25, APRI score ≥1.0 - Any of the following predicting F3-F4 or F4 fibrosis: Fibrosure^TM^ or transient elastography |
| **Treatment Experience** | Receiving any previous HCV therapy, including interferon-based therapy |
| **LTFU Prior to HSSP Referral** | Patient failure to show for follow-up clinic visit and not rescheduling |
| **LTFU After HSSP Referral** | ≥3 attempts to contact the patient by phone and no response to letter mailed to patient’s home for ≥3 months |
| **Patrial Insurance Approval** | 8-week approval of LDV/SOF versus FDA-recommend 12-week duration in patients aged <18 years |
| **Gastric Acid-Reducing Agents** | Any of the following medication classes:   - Proton-pump inhibitor - Histamine type-2 receptor blocker - Antacid |
| **Confirmatory Hepatitis C Testing** | Either of the following:   - Negative antibody >18 months of age - Two consecutive negative HCV RNA results at least 6 months apart |
| Abbreviations: APRI, Aspartate Aminotransferase to Platelet Ratio Index; FIB-4, Fibrosis-4 Index; HCV, hepatitis C virus; LTFU, Lost to Follow-up; HSSP, health system specialty pharmacy; LDV/SOF, ledipasvir/sofosbuvir | |
