# Supplementary material for: Pediatric and Adolescent Hepatitis C Care Cascade and Real-World Treatment Outcomes Utilizing an Integrated Health System Specialty Pharmacy Model
Source: J Pediatric Infect Dis Soc. 2025 May 6;14(5):piaf042. doi: 10.1093/jpids/piaf042 (PMC12123190; doi:10.1093/jpids/piaf042)
Supplement: piaf042_suppl_Supplementary_Table_S3 [file piaf042_suppl_supplementary_table_s3.docx]

Supplementary Table 3. Baseline* Characteristics of Patients with a Clinic Evaluation

|  | 0-5 years *N=42* | 6-11 years *N=41* | 12-17 years *N=15* | Overall *N=98* |
| --- | --- | --- | --- | --- |
| Median age^, years (IQR) | 4 (2.25-5) | 7 (6-9) | 15 (14-16) | 6 (4-9) |
| Male, n (%) ​ | 30 (71%) | 17 (42%) | 3 (20%) | 50 (51%) |
| White, n (%) ​ | 28 (67%) | 31 (76%) | 11 (73%) | 70 (71%) |
| Weight, kg - median (IQR)​ | 17.3 (13-19.8) | 27.7 (24.8-33.4) | 58 (47.9-71.3) | 24.6 (18.3-33.9)​ |
| Insurance type, n (%) ​  Medicaid ​  Commercial ​  None | ​  36 (86%)  4 (10%)  2 (5%) | ​  34 (83%) ​  6 (15%) ​  1 (2%) | ​  12 (80%) ​  3 (20%) ​  0 (0%) | ​  82 (84%)  13 (13%)  3 (3%) |
| Referred to HSSP | 26 (62%) | 33 (80%) | 14 (93%) | 73 (74%) |
| Initiated Treatment | 20 (48%) | 32 (78%) | 12 (86%) | 64 (65%) |
| *Baseline labs were the most recent labs at time of clinic visit.  ^Age at time of clinic visit.  Abbreviations: IQR, interquartile range; kg, kilogram; HSSP, health system specialty pharmacy | | | | |
