# Supplementary material for: Pediatric and Adolescent Hepatitis C Care Cascade and Real-World Treatment Outcomes Utilizing an Integrated Health System Specialty Pharmacy Model
Source: J Pediatric Infect Dis Soc. 2025 May 6;14(5):piaf042. doi: 10.1093/jpids/piaf042 (PMC12123190; doi:10.1093/jpids/piaf042)
Supplement: piaf042_suppl_Supplementary_Table_S4 [file piaf042_suppl_supplementary_table_s4.docx]

Supplementary Table 4. Times Between Stages of the Cascade of Care for Patient Who Initiated Treatment

| Cascade of Care Stage | N* | 3-5 years ​  n^^^=42 | 6-11 years ​  n^^^=41​ | 12-17 years ​  n^^^=15 | Overall ​  n^^^=98 |
| --- | --- | --- | --- | --- | --- |
| Referral to HSSP to Treatment Initiation, median days [IQR] | 64 | 90 [26-190] | 36 [22-127] | 41 [21-90] | 43 [22-129] |
| Referral to HSSP to  Successful Swallowing  Practice, median days  [IQR] | 69 | 35.5 [0-161.5] | 0 [0-9.8] | 0 [0-0] | 15 [5-51.5] |
| Successful Swallowing  Practice to Insurance  Approval, median days  [IQR] | 67 | 15.5 [5.2-62] | 11.5 [4.5-30.2] | 23 [6-53] | 15 [5-51.5] |
| Insurance Approval to  Treatment Initiation,  median days [IQR] | 64 | 12 [7-22] | 11 [7-16] | 13 [10-36] | 11 [7-20] |
| *N is the number of patients completing the designated cascade of care stage  ^^^n is the overall number in the age cohort  Abbreviations: DAA, direct-acting antiviral; IQR, interquartile range | | | | | |
