# Supplementary material for: Pediatric and Adolescent Hepatitis C Care Cascade and Real-World Treatment Outcomes Utilizing an Integrated Health System Specialty Pharmacy Model
Source: J Pediatric Infect Dis Soc. 2025 May 6;14(5):piaf042. doi: 10.1093/jpids/piaf042 (PMC12123190; doi:10.1093/jpids/piaf042)
Supplement: piaf042_suppl_Supplementary_Table_S5 [file piaf042_suppl_supplementary_table_s5.docx]

Supplementary Table 5. Patient-Reported Side Effects

| ​ | **LDV/SOF (n=43)**​ | **SOF/VEL (n=17)**​ | **GLE/PIB (n=4)**​ | **TOTAL (n=64)**​ |
| --- | --- | --- | --- | --- |
| **Patients reporting any SE, n (%)** | 23 (53%)​ | 9 (53%)​ | 2 (50%)​ | 34 (53%)​ |
| **Headache, n (%)** | 9 (21%) | 4 (24%) | 0 (0%) | 13 (20%) |
| **Fatigue, n (%)** | 9 (21%) | 3 (18%) | 1 (25%) | 13 (20%) |
| **Nausea, n (%)** | 4 (9%) | 4 (24%) | 1 (25%) | 9 (14%) |
| **Vomiting, n (%)** | 4 (9%) | 3 (18%) | 1 (25%) | 8 (13%) |
| **Insomnia/Sleep Disturbance, n (%)** | 3 (7%) | 2 (12%) | 0 (0%) | 5 (8%) |
| **Behavioral Changes, n (%)** | 3 (7%) | 1 (6%) | 0 (0%) | 4 (6%) |
| **Appetite Changes, n (%)** | 2 (5%) | 0 (0%) | 0 (0%) | 2 (3%) |
| **Abdominal Pain/Cramps, n (%)** | 2 (5%) | 0 (0%) | 0 (0%) | 2 (3%) |
| **Coagulopathy, n (%)** | 2 (5%) | 0 (0%) | 0 (0%) | 2 (3%) |
| **Dyspepsia, n (%)** | 0 (0%) | 2 (12%) | 0 (0%) | 2 (3%) |
| **Joint Pain, n (%)** | 1 (2%) | 0 (0%) | 0 (0%) | 1 (2%) |
| **Constipation, n (%)** | 1 (2%) | 0 (0%) | 0 (0%) | 1 (2%) |
| **Pruritus, n (%)** | 0 (0%) | 0 (0%) | 1 (25%) | 1 (2%) |
| **Tinnitus, n (%)** | 0 (0%) | 1 (6%) | 0 (0%) | 1 (2%) |
| **HSV Outbreak, n** **(%)** | 1 (2%) | 0 (0%) | 0 (0%) | 1 (2%) |
| Abbreviations: SE, side effect; GLE/PIB, glecaprevir/pibrentasvir; LDV/SOF, ledipasvir/sofosbuvir; SOF/VEL, sofosbuvir/velpatasvir, HSV, herpes simplex virus | | | | |
