# Supplementary material for: Pediatric and Adolescent Hepatitis C Care Cascade and Real-World Treatment Outcomes Utilizing an Integrated Health System Specialty Pharmacy Model
Source: J Pediatric Infect Dis Soc. 2025 May 6;14(5):piaf042. doi: 10.1093/jpids/piaf042 (PMC12123190; doi:10.1093/jpids/piaf042)
Supplement: piaf042_suppl_Supplementary_Figure_Descriptions [file piaf042_suppl_supplementary_figure_descriptions.docx]

Supplementary Figure 1: Flowchart depicting all patients screened and excluded from the study along with reasons for exclusion.

Supplementary Figure 2: Forest plot displaying the results of the ordinal regression

analysis assessing the associations between time from Health-System Specialty

Pharmacy (HSSP) referral to direct-acting antiviral (DAA) initiation and covariates

including ability to swallow, prior authorization outcomes, and medication availability to

HSSP.

Supplementary Figure 3: Bar graph illustrating ability to swallow practice medication at

time of initial evaluation in patients referred to the HSSP (n=73). Subsequent

pharmacist response until successful swallowing was achieved are also depicted.

*Patient contacts occurred during swallowing practice plan. The number of contacts

indicates how many times the pharmacist spoke with the patient or caregiver from

referral until swallowing practice was successful.
